# Wideband Anti-Jamming Based on Free Space Optical Communication and Photonic Signal Processing

**DOI:** 10.3390/s21041136

**Published:** 2021-02-06

**Authors:** Ben Wu, Yang Qi, Chenxi Qiu, Ying Tang

**Affiliations:** 1 Department of Electrical and Computer Engineering, Rowan University, 201 Mullica Hill Rd., Glassboro, NJ 08028, USA; qiy1@students.rowan.edu (Y.Q.); tang@rowan.edu (Y.T.); 2 Department of Computer Science, Rowan University, 201 Mullica Hill Rd., Glassboro, NJ 08028, USA; qiu@rowan.edu

**Keywords:** photonic signal processing, radio frequency photonics, free space optical communication, jamming

## Abstract

We propose and demonstrate an anti-jamming system to defend against wideband jamming attack. Free space optical communication is deployed to provide a reference for jamming cancellation. The mixed signal is processed and separated with photonic signal processing method to achieve large bandwidth. As an analog signal processing method, the cancellation system introduces zero latency. The radio frequency signals are modulated on optical carriers to achieve wideband and unanimous frequency response. With wideband and zero latency, the system meets the key requirements of high speed and real-time communications in transportation systems.

## 1. Introduction

The increasing capacity of wireless networks has revolutionized application scenarios and services deployed in transportation systems [1,2,3,4]. The growing trend of Internet of Things initiatives increasing need of high speed and real-time communications. As our reliance on wireless communication networks increases, cyber-attacks become potentially more disastrous [5,6]. In fact, wireless communication networks, due to their broadcasting nature, are highly vulnerable to jamming attack, which can result in Denial-of-Service (DoS) [7,8,9,10,11].

**Figure 1:**
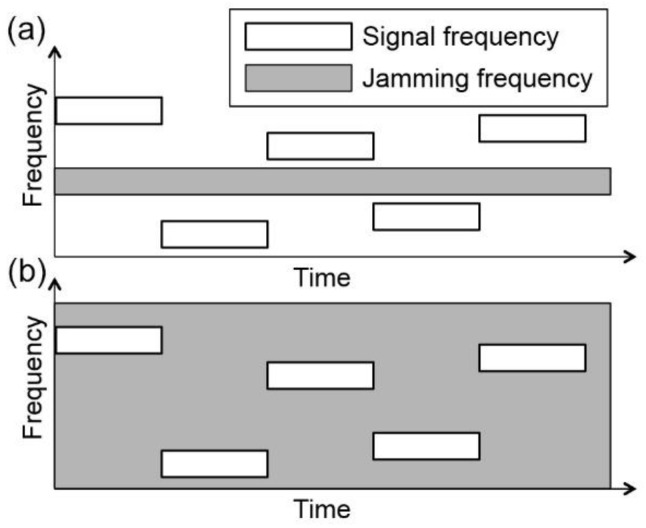
A comparison between (**a**) narrow band jamming (**b**) wide band jamming.

Most of the existing anti-jamming methods fall into two categories, spread spectrum-based techniques and jamming cancellation techniques [12,13,14,15,16]. Spread spectrum-based anti-jamming techniques rely on pre-shared codes between legitimate transmitters and receivers. For example, as a widely used spread spectrum-based anti-jamming technique, Frequency Hopping method changes the signal carrier frequency based on the pre-share codes, so the jammer cannot follow the change [17,18,19,20,21,22]. This method is only effective for narrow band jamming. If the jamming spectra cover all possible frequencies, there is no clear band to switch to (Figure 1). Similarly, as another spread spectrum-based anti-jamming technique, Direct Sequence Spread Spectrum (DSS) multiplies the original signal with pseudo random noise code to spread the signal spectrum at the transmitter and recover the spectrum at the receiver [23,24,25]. If the jamming signal has a wide band spectrum with high power, the signal to noise ratio (SNR) of the recovered signal of interest (SOI) is also seriously affected by the jamming signal.

The jamming cancellation technique provides an active way to defend jamming attacks. It removes the jamming signal and extracts the SOI at the receiver. Traditional jamming cancellation techniques are based on processing the mixture of jamming signal and SOI in a digital way. Since the malicious jamming signal is random, there are very few hints for this blind source separation (BSS) process [26,27]. The emerging technique of multiple-input multiple-output (MIMO) provides some hints. By using multiple antennas at the receiver, the jamming signal and SOI can be differentiated by the positions of the jammer and legitimate transmitter [28,29]. Even with the help of MIMO, to separate the wide-band jamming signal from the SOI is still challenging. To separate SOI from the jamming signal in a MIMO system requires analog to digital conversion (ADC) and digital signal processing (DSP) for all the antennas. If the jamming bandwidth is in the range of GHz, it is extremely difficult for the mobile devices to achieve ADC in such bandwidth [30,31]. Moreover, jamming cancellation based on DSP is a multi-variable process, and the difficulty of digitally solving this multi-variable process increases with the bandwidth of the signals.

**Figure 2:**
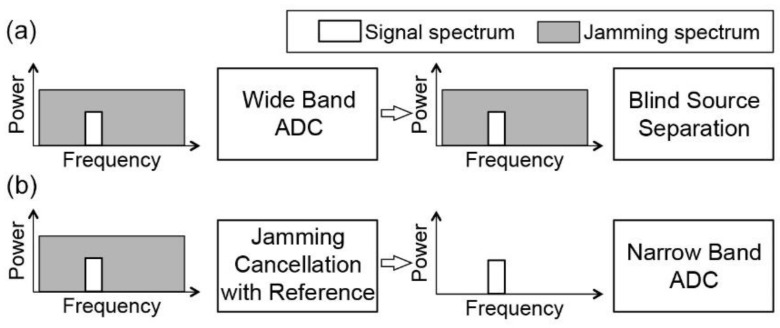
A comparison between (**a**) digital jamming cancellation and (**b**) analog jamming cancellation.

In this paper, we solve the wide band anti-jamming problem by using a bandwidth independent method to remove the jamming before ADC, and introduces a reference signal for the jamming separation, so the multi-variable problem can be simplified, and each variable is solved separately. The bandwidth independent method is photonic signal processing, which processes and cancels the wide-band jamming signal in the analog domain, so narrow-band ADC can be applied to the SOI (Figure 2). The reference signal for jamming separation is transmitted through a free space optical (FSO) communication channel. Since the bandwidth of a FSO channel is up to 20 GHz [32,33] a single FSO is able to carry reference signals for canceling jamming signals that covers all the possible RF communication bands. The FSO channel carries both of the signal of interest and the jamming signal, and by using the protocols and network model discussed in Section 3, the receiver is able to cancel the jamming signal with the FSO reference signal. Since the optical carriers have much higher frequencies than the radio frequency carriers, the bandwidths of the optical carriers are much larger than the radio frequency counterpart. By properly choosing the FSO transmitter, as is discussed in Section 4, the wideband property of the FSO channels can be fully utilized, so jamming signals with GHz bandwidth can be canceled in real-time. Since the FSO has been widely deployed in the transportation systems [34,35,36], the anti-jamming system can be easily implemented with the existing transportation infrastructures with relatively low cost.

## 2. Background 

The jamming cancellation system deploys the emerging photonic signal processing technique to cancel the jamming signal and free space optical communication technique to provide a reference signal for the cancellation. This section discusses the features of photonic signal processing and free space optical communication, and shows that both of the wideband signal processing and high speed data transmission feathers improve the performance of a jamming cancellation system.

### 2.1. Photonic Signal Processing

Photonic signal processing processes analog signals directly without ADC [37,38]. One of the advantages of optics and photonics-based methods is large bandwidth [39,40]. The optical carrier frequencies are much higher than both RF baseband signals and RF carrier frequencies. The carrier frequency for c-band optical communication is 193THz, which is at least four orders of magnitude larger than the maximum frequency of most RF signals in the 4th generation (4G) networks [41]. The large carrier frequencies enable unanimous frequency response in GHz bandwidth, which means optical and photonic devices can process signal with GHz bandwidth. Another advantage is the low latency [42,43]. Low latency is achieved not only because the time consumption of wide-band ADC is saved, but also based on the fact that the photonic methods process the signals in a different mechanism compared with DSP. The signal is processed by the propagation of light waves in resonator, modulators, amplifiers, nonlinear fibers, and, etc., or in another word, at the speed of light [44]. While in DSP, latency is introduced because of the limit of the processor clock, and latency increases exponentially with the bandwidth and the power of the interference to be processed. By using the photonic method to process the analog signals, signal of interested is separated from the wideband interference, so narrow band and low-resolution ADC is needed at the receiver. Compared with digital method that requires wide band and high-resolution ADC, the photonic method scientifically reduces the latency. 

A wide range of signal processing functions has been studied with the optics and photonics-based methods. For example, logic Exclusive OR (XOR) has been demonstrated by semiconductor optical amplifier [45,46]. The function of optical thresholder is achieved by nonlinear optics [47,48]. Weight control for multiple signals has been achieved by using the attenuation of waveguides [49,50]. 

The wide bandwidth and zero latency properties of photonic signal processing are especially important for anti-jamming. The performance of an anti-jamming system is measured by its operational bandwidth and cancellation depth. As DSP methods typically work under Nyquist–Shannon sampling theorem, the operational bandwidths of DSP-based anti-jamming methods are limited by the ADC circuits. Moreover, with wideband jamming signals (Figure 2a), power consumption of ADC and DSP circuits increase exponentially with the bandwidth, and considerable power consumption and latency are introduced if the bandwidth of the jamming signal is in GHz range. By using photonic-based methods to pre-process the analog signal before ADC, the problem of digitizing and processing a wide band signal can be simplified to a narrow band signal processing (Figure 2b). The time and power consumption of DSP can be reduced by orders of magnitudes. Table 1 summarize the comparison of the anti-jamming techniques. The spread spectrum techniques are effective when the jamming signal has a narrow bandwidth [17,18,19,20,21,22]. For wide band signals, digital jamming cancellation method introduces large latency, and the photonic method has less latency [42,43]. As an analog signal processing method, the photonic method is also scalable to multiple stages, and the cancellation ration can be multiplied by the stages.

The photonic signal processing provides instant jamming cancellation, and is compatible with the existing spectrum sensing methods that detect the existence of the jamming signals [51,52,53]. By using the learning algorithm to improve the spectrum sensing, the jamming signal can be detected in real-time [54,55,56]. With the data from the spectrum sensing, both of the spectrum resources and the jamming cancellation resources can be allocated based on the existence of the jamming signal and the needs for communication bandwidth [57].

### 2.2. Free Space Optical Communication

FSO has been widely studied as an alternative solution for wireless communications. Hybrid FSO/RF network has been proposed for the next generation wireless communications (fifth generation (5G) networks) [58,59,60]. In this paper, an FSO transmitter functions as an anti-jamming station that receives the RF jamming signal, modulates the RF jamming signal to a FSO channel and sends the FSO signal to the legitimate receivers. The carrier frequencies of the FSO channels range from 192THz to 750 THz. Compared with RF wireless communication carrier frequencies that range from 500 kHz to 6GHz (fourth Generation wireless network) and 30 GHz to 70 GHz (5G), the bandwidth of a FSO channel is orders of magnitude larger than a RF channel. The disadvantages of FSO channels are the large power consumption at the transmitter, and high directionality, which requires the line-of-sight transmission. Because of such disadvantages, FSO cannot completely replace RF communications, and mostly exists in a hybrid FSO and RF communication system.

This paper strategically exploits both the advantages and disadvantages of the FSO channel. First, the large bandwidth of the FSO channel can provide the reference for removing the jamming signal that covers all the possible RF bands. Second, the high directionality of the FSO channel, which is normally considered a limitation of FSO, can in fact be used to benefit the anti-jamming system: the line-of-sight requirement ensures the high spatial selectivity between the FSO transmitter and FSO receiver, which protects the FSO channel itself from being jammed. If the jammer sends out a strong optical beam to blind the legitimate FSO receiver, the receiver can select to receive optical signals from the legitimate FSO transmitter by using a lens hood. The position of the FSO transmitter needs to be carefully selected for the receiver to differentiate the jammer and the FSO transmitter and a handshaking protocol is needed between the transmitter and receiver, which is discussed in Section 3. Third, since the FSO signal is carried by a light beam generated with lasers, the power consumption of FSO transmitter is always an issue for battery powered devices. This hybrid system does not simply turn off RF communications, and switches to FSO communications when the RF channels are being jammed. The FSO channels are used to provide reference signals for jamming cancellation in the RF channels. The battery-powered devices only receive the optical beams, which requires relatively less power than sending the optical beams. The FSO transmitters are immobile and powered by cable. 

## 3. Methods and System Model

**Figure 3:**
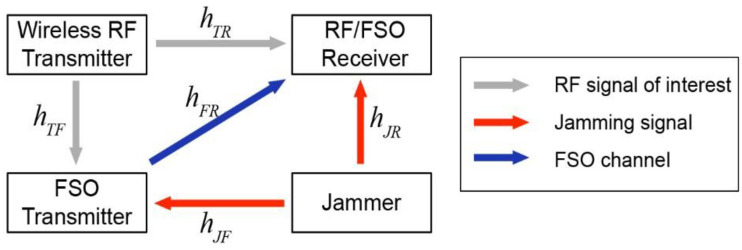
Schematic diagram of the anti-jamming system. The free space optical (FSO) transmitter receives both of the signal of interest from the wireless RF transmitter (grey arrow) and the jamming signal from the jammer (red arrow). The FSO transmitter modulates both of the signals on optical carriers, and sends the signals to the FSO receiver through FSO channel (blue arrow).

Figure 3 shows the schematic diagram of the anti-jamming system. The wireless RF transmitter and receiver communicate with RF channels. The jammer sends out RF jamming signals that overlap with the legitimate RF bands for the transmitter and the receiver. The FSO transmitter functions as an anti-jamming station that receives the jamming signal and modulates the RF signal on to an optical carrier and send it to the receiver through an FSO channel.

The FSO transmitter receives both jamming signals and SOI from legitimate transmitter. The relative amplitude of the jamming signals and the SOI received by FSO transmitter depends on relative positions of the FSO transmitter, jammer, and legitimate transmitter, and can be controlled by selecting the appropriate FSO transmitter. A mixture of SOI and jamming signals are received at the FSO transmitter and the mixed signals are modulated to the FSO channel. The RF/FSO receiver receives both RF signals *r_rf_* and FSO signals *r_fso_*, which can be expressed in the following equation:

(1) $$\left[\begin{array}{c} r_{rf}\\ r_{fso} \end{array}\right]=\left[\begin{array}{cc} h_{1} & h_{2}\\ h_{3} & h_{4} \end{array}\right]\left[\begin{array}{c} s_{soi}\\ s_{jam} \end{array}\right]$$

where *s_soi_* is the SOI, *s_jam_* is the jamming signal, and *h_i_*, where *i* = 1,2,3,4 is the complex channel coefficient. The channels can be either RF channels or FSO channels. *h_i_* is a complex number. The signal attenuation/amplification is the amplitude of *h_i_*, and the signal phase delay is the phase of *h_i_*. Using the channel coefficients shown in Figure 3, Equation (1), can be written in the following form.

(2) $$\left[\begin{array}{c} r_{rf}\\ r_{fso} \end{array}\right]=\left[\begin{array}{cc} h_{TR} & h_{JR}\\ h_{TF}h_{FR} & h_{JF}h_{FR} \end{array}\right]\left[\begin{array}{c} s_{soi}\\ s_{jam} \end{array}\right]$$

**Figure 4:**
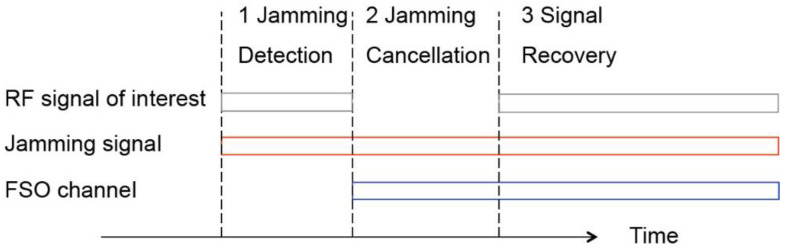
System response to jammers.

To separate the jamming signal and the SOI, we need to solve Equation (1) from the received signals *r_rf_* and *r_fso_*. This is a complex BSS problem, and considering both phases and amplitudes of *h_i_*_=1,2,3,4_, there are 8 unknown parameters for the optimization process. In this system, the blind source problem is simplified to a noise cancellation problem by properly designing the protocols for anti-jamming. As shown in Figure 4, when the jamming is detected by the FSO transmitter, a command is sent to the RF transmitter/receivers through FSO channel to switch the system to the anti-jamming mode. At the step of jamming cancellation, SOI is turned off, which means *s_soi_* = 0, and Equation (2) is simplified to

(3) $$r_{rf}=h_{JR}s_{jam}$$

(4) $$r_{fso}=h_{JF}h_{FR}s_{jam}$$

Equation (3) shows the jamming signals, and Equation (4) shows the reference signals. Both the jamming signal and reference signals are wide band signals, and are feed into the photonic signal processing circuit. The photonic signal processing circuit has two functions: first, to match the channel coefficient (Equation (5)); second, to subtract the received jamming signal by the reference signal (Equation (6)).

(5) hM=hJRhJFhFR

(6) $$r_{rf}-h_{M}r_{fso}=0$$

where *h_M_* is to match the amplitude and phase of jamming signal channel coefficient *h_JR_*, and reference signal channel coefficient *h_JF_h_FR_*. *h_M_* is achieved in the photonic circuits by controlling the amplitude and phase of the optical signal. This is an optimization process of finding the minimum of the residue the of jamming signal *r_rf_ − h_M_r_fso_* by changing the phase and amplitude of the reference signal to match with the jamming signal.

Once the matching condition is satisfied, the SOI can be switched on. This is the signal recovery step in Figure 4. The output of the cancellation system is

(7) $$r_{out}=r_{rf}-h_{M}r_{fso}=h_{TR}s_{soi}-h_{M}h_{TF}h_{FR}s_{soi}$$

The first term is the direct transmission from the legitimate transmitter and receiver, and the second term is the interference generated from the reference signal. This interference is similar to the multi-path problem in wireless communications. The *r_out_* is a narrow-band signal, and SOI can be recovered from rout with narrow band ADC and signal processing circuits.

## 4. System Validation

### 4.1. Results

**Figure 5:**
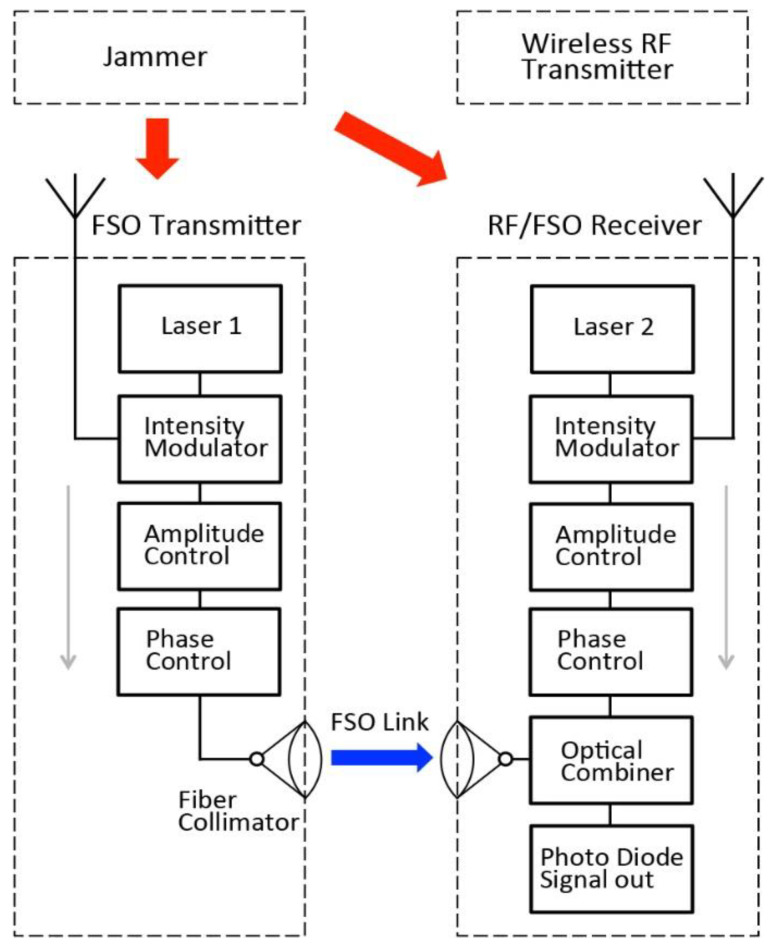
Experimental setup of the anti-jamming system.

Figure 5 shows the experimental setup, which corresponds to jammer, FSO transmitter, RF/FSO receiver in the schematic diagram (Figure 2). The FSO transmitter is able to detect and receive the RF jamming signals and modulate the jamming signal on to a laser carrier (Laser 1). The modulated signal is sent through a FSO link to the RF/FSO receiver. RF/FSO receiver receives both the RF signals and FSO signals for jamming cancellation. RF signals are modulated to another laser carrier (Laser 2). Both the FSO transmitter and RF/FSO receiver is able to control and match amplitude and phase of the modulated optical signal to achieve matching condition, which is to solve *h_M_* in Equation (6). 

The optical wavelength for the FSO transmitter (Laser 1 in Figure 5) is 1544 nm, and the optical wavelength for jamming cancellation at the FSO receiver (Laser 2 in Figure 5) is 1560 nm. We choose the wavelength at the optical communication band, so the corresponding optical components, such as optical amplifiers, attenuators, modulators, and delays, are available at this wavelength range. The amplitudes of the optical signals at the FSO transmitter and the receiver are controlled by the Erbium-doped fiber amplifiers (EDFAs) and tunable optical attenuators. The phases of the optical signals at the FSO transmitter and the receiver are controlled by the tunable optical delays.

Once the jamming signal is detected, the FSO transmitter is turned on and the system operate at step 2 in Figure 4. The intensity modulator in FSO transmitter is inversely biased to achieve the cancellation function. An optical combiner is used to combine the modulated signal at the RF/FSO receiver, and the reference signal from the FSO link. The photodiode at the receiver converts the combined optical signal to RF signal. At phase 2, feedback control is applied to minimize the output power and achieve cancellation. 

**Figure 6:**
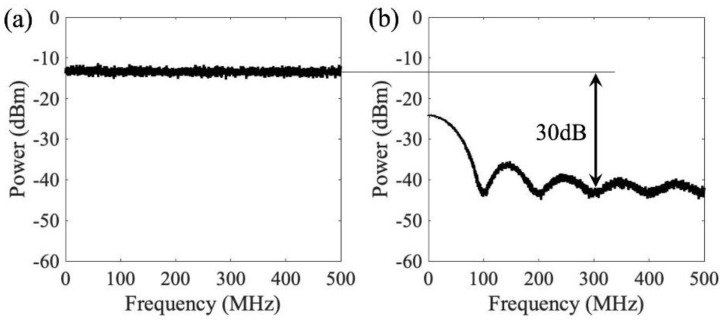
Base band spectrum of anti-jamming system (**a**) Received signals without removing the jamming signal (**b**) Recovered signal of interest after removing the jamming signal.

Figure 6 shows the cancellation results at the receiver in phase 2. The jamming signal is random white Gaussian noise with bandwidth of 1 GHz, and the signal of interest is binary polar non-return to zero signal with 100 Mbps data rate. Figure 6a is the base band spectrum of signal received at the receiver without removing the jamming signal. Both of bandwidth and power of jamming signal are at least one order of magnitude larger than the signal of interest. The mixed spectrum (Figure 6) only shows the white gaussian noise, and does not shows the pattern of the signal of interest. Figure 6b shows the recovered signal of interest after removing the jamming signal; 30 dB cancellation is achieved over the jamming signal bandwidth, and the pattern of the signal of interest is clearly shown. In the system test, the jamming signal and signal of interest are digitally simulated signal and the 30dB cancellation ratio is based on experimental test result. To measure the cancellation ratio, a network analyzer (Keysight E5063A) is used; 30 dB of cancellation ratio is measured by turning off the signal of interest and feeds the signal output of the network analyzer to both of the RF input of the FSO transmitter and the receiver. The output of the receiver photodiode shows a 30 dB cancellation. To minimize the effect of antenna with limited bandwidth, the test result is obtained by replacing the wireless channels between the jammer, FSO transmitter, and RF/FSO receiver with RF cables and RF splitters. 

Figure 6 shows the jamming cancellation for baseband signals. The wideband properties of the photonic jamming cancellation method enable the system to process signals with radio frequency carriers, or in another word, bandpass signals. In such cases, the jamming signal and the signal of interest has the same carrier frequency and are overlapped in the radio frequency spectrum. Since the processing bandwidth of the system is higher than the radio carrier frequency, signal of interest with multiple channels can be processed simultaneously with one photonic system. 

### 4.2. Network Implementation

**Figure 7:**
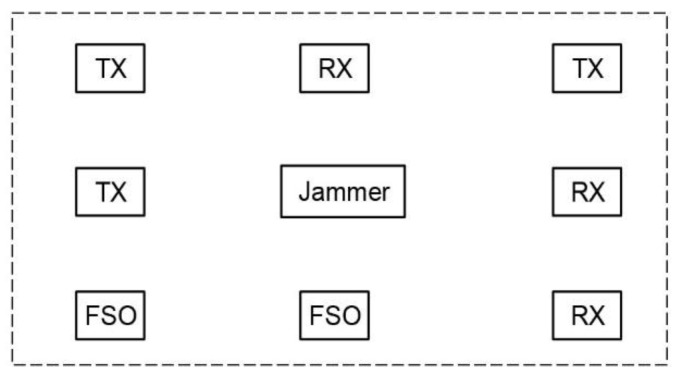
Anti-jamming network: the rectangles labeled with “FSO” in the bottom left and bottom middle show two FSO transmitters (TX: wireless RF transmitter, RX: RF/FSO receiver, FSO: FSO transmitter).

The jamming attacks can be identified by measuring the availability of the wireless communication channels; however, the antennas and signal processing units of the legitimate users have limited bandwidth, and the detection of jamming based on the each users is lack of overall control of all the RF bands under attacked. Moreover, if all the RF bands are being jammed, the users and controller cannot communicate to send commands in anti-jamming protocol. FSO transmitters behave as anti-jamming stations and have spectrum sensing facilities to detect the jamming attacks. The FSO transmitters are powered by cables and mobile users are powered by batteries. The FSO transmitters have more power budget and can be equipped with RF spectrum analyzers, and measure the wireless signal with a wider bandwidth and faster measuring rate. Multiple FSO transmitters exist in anti-jamming network. Figure 7 shows an example of anti-jamming network, where two FSO transmitters are located in the bottom left and bottom middle of the figure. The number FSO transmitters is scalable to *n* based on the size of the network and locations of the RF receivers. of The FSO transmitters measure the RF spectrums at different positions and shared the measured results with each other to determine whether a wide-band jamming attack has been identified and the network should be switched to the anti-jamming mode.

The position of the jammer determines the channel coefficients *h_JR_* and *h_JF_*, and pre-known information about the channel coefficients can greatly simplify the process of finding the matching condition and canceling the jamming (Equations (5) and (6)). Moreover, the relative positions between FSO transmitters, jammer, and legitimate transmitters/receivers determine of the performance of the system and the SNR of the recovered signal. As an extreme example, if the FSO transmitter is close to the legitimate user or the jammer and legitimate transmitter are at the symmetric positions to the FSO transmitter, the channel coefficients in Equation (7) satisfy:

(8) $$h_{TR}-h_{M}h_{TF}h_{FR}\approx 0$$

The output of the cancellation system is *r_out_* ≈ 0, and the SNR of the recovered SOI is also close to zero. The position of the jammer can be estimated by the multiple FSO transmitters. The multiple FSO transmitters function as a phase array antenna, and jammer localization can be approximately measured by the relative phase and amplitude of the jamming signal detected by each FSO transmitter.

Generally, the closer distance between the FSO transmitter and the legitimate receiver, the closer values of the two terms in Equation (8), and the smaller SNR of the recovered signal. This issue can be addressed by properly selecting the FSO transmitter to serve for a certain user. The flexibility of selecting the FSO transmitter is another advantage of the hybrid system compared with the jamming separation methods based on MIMO techniques. The counterparts of the FSO transmitter and the legitimate receiver are two antennas in a MIMO system. In a MIMO system, the relative distance between the two antennas is limited by the size of the mobile device, and thus the spatial resolution is also limited. In this hybrid system, there is no distance limit between FSO transmitter and legitimate receiver.

**Figure 8:**
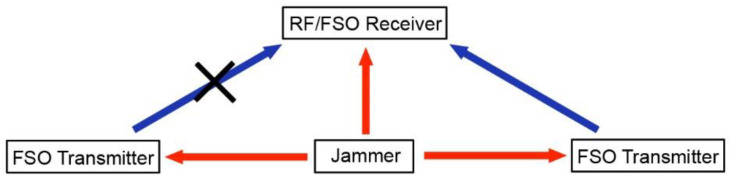
Switch from a blocked FSO channel to an unblocked FSO channel.

The selection of the FSO transmitter also depends on the visibility of the FSO transmitter to the legitimate receiver. Because of the high directionality of the FSO channel, an optical beam may be blocked from the line-of-sight of the receiver. In this case, the receiver needs to switch to another FSO transmitter and rebuild the matching condition (Figure 8). The dynamic selection of FSO transmitter also ensures the FSO channel to maintain its wide-band transmission, so jamming signals with GHz bandwidth can be cancelled consistently. 

## 5. Conclusions

We proposed and demonstrated an anti-jamming system for wide-band jamming cancellation. Both the network model and the physical layer implementation are studied. The jamming cancellation is based on photonic signal processing, which processes GHz signal with zero latency. FSO channel is used to provide reference signal to cancel the jamming signal. The separation of FSO transmitter and the legitimate user enable large angle resolution that differentiate the legitimate transmitter and the jammer. Wide band and real-time signal processing enables the deployment of the hybrid system in transportation networks that require both high speed and instant communication. The main contribution of this work is to use optical spectrum to carry the reference signal and remove the jamming signal with the optical reference signal. Compared with the similar concepts from radio frequency counterpart, the system in this manuscript processes the signals on optical carriers and achieves a much larger bandwidth. The prospective work will implement the anti-jamming system on both the existing 4G and evolving 5G networks. The wideband property of the photonic system meets the requirement of high data rate of 5G network.

## Figures and Tables

**Table 1:** Comparison of different anti-jamming techniques. ADC: analog to digital conversion; MIMO: multiple-input multiple-output; FSO: free space optical communication.

Description	Spread Spectrum-Based Techniques	Digital Jamming Cancellation	Photonic Jamming Cancellation
Jamming Signal Bandwidth	Narrow	Wide, introduce large latency	Wide, low latency
Cancellation ratio	Not applicable	Depend on ADC resolution	Scalable, can be multiplied with multiple stages
Latency	Low	High	Low
Hardware requirement	Spectrum sensing	MIMO system	FSO system

## Data Availability

The data presented in this study are available on request from the corresponding author.
